# Thomas Jefferson and the University of Virginia

**Published:** 1889-09

**Authors:** 


**Thomas Jefferson and the University of Virginia.** By H. B. ADAMS, Ph. D. Volume No. 2.

This is one of a series of volumes issued by the United States Bureau of Education, giving the history of educational institutions. No. 1 was largely devoted to the history of the College of William and Mary, of Virginia, and the current, No. 2, is principally devoted to the scheme of Jefferson for the founding of the University of Virginia; and it certainly presents a pleasant picture of the Virginia statesman in his efforts for higher education in his own State. He was hardly behind the New England sentiment, which favored State aid for higher education, and local taxation for support of primary schools. For fifty years he struggled against the selfishness of the wealthy class in behalf of the "holy cause of the university," and to this day Jefferson is the foremost figure of the promoters of educational interests in Virginia. During his diplomatic residence in France he made a study of European universities, and he caught the French spirit in the educational sphere quite as much as in the political.

It is remarkable that when France lost her territorial influence and control in the West, some of the leading spirits made earnest efforts to impress upon the United States, French thought and French educational methods through a Southern university. The story in the volume reviewed, of Chevalier Quesnay's project of a university under the patronage of Jefferson and other Virginia leaders, reads like a romance. He was the grandson of the famous court physician of Louis XV., and served for some time in our army of the revolution.

It was to be a French academy he would found, to be equipped with French professors. Richmond was to be its seat. Its cornerstone was laid June 24, 1786. Quesnay then returned to France to complete his plans for an intellectual and educational union between France and the United States. This was at a period when Rousseau and the Encyclopediasts dominated French thought, and there was great danger that they become potential influences in the Southern States. But that influence was checkmated in time by a current of Scottish Presbyterianism proceeding from Princeton College.

Jefferson's bill of 1779 provided for the foundation of common schools for both male and female children, ten years in advance of the time when even Boston gave a place to female children in her public schools. In connection with his system was a system of township government and taxation, after the type of that of New England, whose power he felt in the hostility of New England to his own policy when at the head of our government. Referring to this concentrated power in townships, and to its energy at the time of the Embargo, Jefferson said he "felt the foundation of the government shaken under his (my) feet by the New England townships." Quesnay's plan did not mature, and it is very remarkable that, in 1794, the French faculty of the College of Geneva, Switzerland, proposed to Jefferson to transfer that college to Virginia. Jefferson favored it, and endeavored, unsuccessfully, to influence Washington to second the scheme; but the Virginians did not sustain Jefferson in this project. And it is well, for it was far better for American institutions to represent the American spirit, than to start under the auspices of French philosophers. Jefferson was, as is well known, an advanced liberal in religion, yet it is matter of interest to find that he favored placing the ethical education of children upon a theistic basis. He says: "The proofs of the being of a God, the creator, preserver, and supreme ruler of the universe, the author of all the relations of morality, and of the laws and obligations, these questions will be within the province of the professor of ethics." He even favored the establishment, in the immediate vicinage of the university, of theological classes by different sects, which he thought would create a spirit of toleration, "and make the general religion a religion of peace, reason, and morality." Here he anticipated, in large degree, the policy of several of our leading universities. It certainly is pleasing to see the intense democratic leader of American politics, who represented all the bitterness in controversy characteristic of the early period of the Republic, devoting his old age, as well as his early years, to the promoting that higher education which is a true glory to a commonwealth, and to see his early philosophic hardness—for such it was—softening as he advanced in years, until toleration and charity and social "sweetness and light" chastened and subdued all the harsher elements of his nature.

Much of the volume we have noticed treats of the influence and power of the University of Virginia, which the author of that paper regards the transcendent intellectual influence in the South.

It closes with a review of other Virginia colleges, their growth and their present character.

A continuation of this series, which shall embrace the higher educational history of the whole country, presented with equal intelligence and breadth, as appears in the volume before us, will be a valuable contribution to the literature of the country.

